# Outcome of Late Presentation of Posterior Urethral Valves in a Resource-Limited Economy: Challenges in Management

**DOI:** 10.1155/2012/345298

**Published:** 2012-09-19

**Authors:** Odutola Israel Odetunde, Oluwatoyin Arinola Odetunde, Adesoji Oludotun Ademuyiwa, Henrietta Uche Okafor, Uchenna Ekwochi, Jonathan Chukwuemeka Azubuike, Nene Elsie Obianyo

**Affiliations:** ^1^Department of Pediatrics, Enugu State University Teaching Hospital, Enugu 400261, Enugu State, Nigeria; ^2^Pediatric Nephrology Unit, Department of Pediatrics, University of Nigeria Teaching Hospital, Ituku Ozalla 402108, Enugu State, Nigeria; ^3^Pediatric Surgery Unit, Department of Surgery, Enugu State University Teaching Hospital, Enugu 400261, Enugu State, Nigeria; ^4^Pediatric Surgery Unit, Department of Surgery, College of Medicine, University of Lagos, Idi Araba 100254, Lagos, Nigeria; ^5^Pediatric Surgery Unit, Department of Surgery, University of Nigeria Teaching Hospital, Ituku Ozalla 402108, Enugu State, Nigeria

## Abstract

Delayed presentation of patients with posterior urethral valve with complications like severe urosepsis, uremia, and anemia are seen in our setting. Renal replacement therapy which should have been offered to these patients is not readily available for children in our country. The aim of this study is to determine the pattern of late presentation and outcome of management of posterior urethral valve in a resource-limited setting. A descriptive retrospective study (1997–2009) was conducted. Data including pattern of presentation, duration of symptoms, complications, and outcome of initial management were analyzed. Twenty-one patients were seen. The median age was 3 years (2 days–13 years). The mean duration of symptoms before presentation was 2.6 years. Nineteen patients (91%) presented with urosepsis while 8 patients (36%) presented with significant renal insufficiency. Laboratory findings varied from-mild-to marked elevation in serum creatinine. Radiological findings confirmed the diagnosis of posterior urethral valve. We concluded that late presentation is common in our setting. This is associated with high morbidity and mortality rates. Efforts at improving awareness and early diagnosis among the health team should be made to stem the tide.

## 1. Introduction

Posterior urethral valves (PUV) are the commonest cause of lower urinary tract obstruction in male infants [1–3]. The incidence of this congenital anomaly in our setting is unknown, although reports from Unites States and Europe indicates that it occurs in about 1 : 8000 and 1 : 25,000 male live births [1, 3, 4]. 

Prolonged and unrelieved lower urinary tract obstruction leads to back pressure effects on the kidneys resulting in obstructive uropathy with renal impairment [5]. PUVs are also a common cause of chronic renal failure in children if treatment is delayed [6–8]. Late presentation in patients with PUV is associated with urosepsis, uremia, and anemia and these form the bulk of patients seen in our centre. 

Early diagnosis and prompt commencement of treatment is therefore germane to the overall outcome of these patients [9, 10]. This is particularly important in a resource—limited environment like ours where facilities for renal replacement therapy in children is not readily available. The aim of this study is to document the pattern of presentation in our centre and outcome of management.

## 2. Materials and Methods

This is a retrospective descriptive study. Records of patients with PUV at the University of Nigeria Teaching Hospital (1997–2004) and Enugu State University of Technology Teaching Hospital, both in Enugu from [2005 to 2009] were reviewed. Inclusion criteria were all patients who had radiological diagnosis of PUV with voiding cystourethrogram and renal ultrasound. Late presentation is defined in this study as patients presenting to our centre four or more weeks after the onset of symptoms. Data such as age at presentation, symptoms and duration of symptoms, complications, investigation, and initial management instituted were collated. Patients were managed between the pediatric nephrology and pediatric surgical units. Data was analyzed using Statistical Package for the Social Sciences version 11.0.

## 3. Results

**Figure 1:**
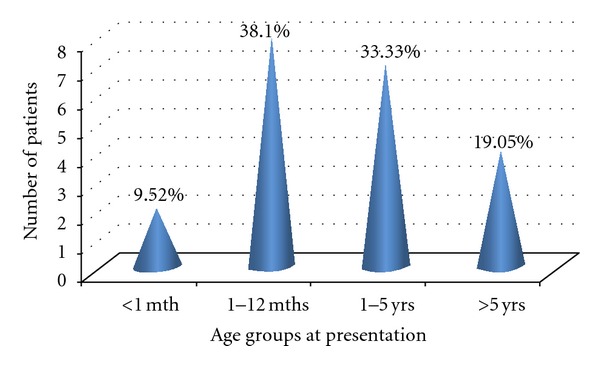
Age distribution of patients at presentation.

There were 21 patients. They were all boys. The age ranges from 2 days to 13 years (Figure 1). The patients mean age at presentation is 2.75 + 3.67 years while the mean duration of symptoms is 2.59 + 3.56 years.

Clinical findings at presentation include voiding anomaly (100%); recurrent fever (90.5%); ballotable kidneys (71.4%); palpable bladder (76.2%); failure to thrive (28.6%); other symptoms like urinary incontinence, polyuria, and enuresis (47.6%) (Table 1).

**Figure 2:**
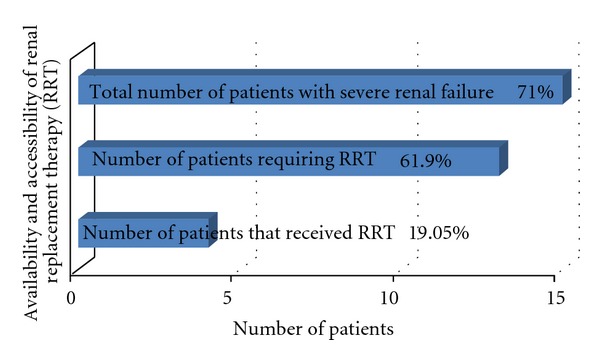
Patients with severe renal failure and required renal replacement therapy (RRT) at presentation.

 Complications at presentation were renal failure (71.4); urinary tract infection (90.5%); anemia (57.2%); high blood pressure (47.6%). *Escherichia coli *accounted for 66.7% of the urinary tract infection (UTI) while 14.3% did not grow any organism. *Pseudomonas aeruginosa* was found in 4.8% of the urine culture of patients with UTI while 14.3% also grew *Klebsiella*. Only one (4.8%) of the patients had vesicoureteral reflux on the voiding cystourethrography. Seventy-one percent of the patients had severe renal failure at presentation. Of these, 62% required renal replacement therapy but only 19.05% were able to receive the therapy during the study period (Figure 2).

Initial management instituted were continuous bladder drainage by urethral catheterization in 76.2% and cutaneous ureterostomy in 23.8% and none had vesicostomy.

The outcome of the patients showed that 31% survived on dialysis (23% received peritoneal dialysis and 8% received hemodialysis). The remaining 69% were lost to followup or presumably died for inability to access required management for profound renal impairment.

## 4. Discussion 

Posterior urethral valve is the commonest cause of obstructive uropathy in children [11, 12]. Posterior urethral valves and other congenital obstructive uropathy accounted for 1.4–6.4% of all renal diseases in most of the centers in Nigeria [13–15]. All (100%) our patients presented with one form of voiding abnormality or another. Most of them presented with poor urinary stream while some others presented with straining during micturition. A few also presented with overflow incontinence. From the foregoing, it is important to have a high index of suspicion of PUV in any child presenting with the above symptoms and refer to a specialist early for appropriate intervention. 

Furthermore, most of our patients presented late with features such as ballotable kidneys and palpable bladders. While the diagnosis of PUV can be made prenatally [16–18], none of our patients was diagnosed prenatally. Negative attitude, long distances to service providers, considerably heavy financial cost, long waiting periods, and unsatisfactory previous scan experience are major barriers to prenatal ultrasound in a study from Nigeria [19], and these barriers had indirectly made prenatal diagnosis of PUV intricate in our setting. The presence of ballotable kidneys suggests back pressure effect of the lower urinary obstruction with attendant hydronephrosis. This predisposes to stasis of urine and colonization by bacteria with attendant urinary tract infection and fever which was present in over 90% of our patients. 

In addition, majority of our patients also presented with complications including high blood pressure, urosepsis, and renal failure. Elevated serum creatinine level has been shown to be associated with poor prognosis in PUV patients [20–22], and this may not be reversed even with the relief of the obstruction. 

The reasons for delayed presentation among our patients are unknown. However, it may not be unconnected with pervasive poverty prevalent in the population. Parents may not seek medical care due to financial ineptitude. Another possible reason is the ignorance that a poor urinary stream could be a transient event and that the baby will improve with time. Lastly, the healthcare poor referral system could be a cause of late presentation. A situation in which general practitioners do not refer patients to specialist for early diagnosis of the cause but rather they treat only the symptoms should be discouraged. The maxim should be that “any child presenting with poor urinary stream should see a specialist for a second opinion”.

Early presentation in a setting such as ours enables early diagnosis and intervention. This reduces the incidence of complications. Several studies have shown better preservation of renal function with early intervention and relief of obstruction [9, 23, 24]. Therefore, with early presentation, diagnosis, and treatment the outcome is expected to improve.

The survival figure of 31% in this study is low compared to other centers in Africa (87.5%) and western world (96.2–100%) [25–28]. There is also a poor system of followup in our setting. These are pointers that require urgent attention. Medical education programs to improve the awareness among general practitioners should be encouraged by the Health Ministry. Mothers presenting with oligohydramnious to the obstetricians should have their neonates screened for PUV when born. Patients presenting with features of urinary tract infection should have a full workup to rule out underlying urogenital anomaly. 

In conclusion, this study has shown that there is delayed presentation of patients with PUV in our setting. This is associated with high morbidity and mortality rates. Efforts at improving awareness and early diagnosis among the health team should be made to stem the tide. 

## Figures and Tables

**Table 1:** Age distribution of patients and clinical features at presentation.

Age group	*n* (%)	Voiding abnormality	Recurrent fever	Ballotable kidney	Palpable bladder	Failure to thrive	Others
<1 month	2 (9.5)	2 (9.5)	0 (0.0)	1 (4.8)	1 (4.8)	0 (0.0)	0 (0.0)
1 month–1 year	8 (38.1)	8 (38.1)	8 (38.1)	3 (14.3)	4 (19.1)	0 (0.0)	0 (0.0)
1–5 years	7 (33.3)	7 (33.3)	7 (33.3)	7 (33.3)	7 (33.3)	3 (14.3)	6 (28.6)
>5 years	4 (19.1)	4 (19.1)	4 (19.1)	4 (19.1)	4 (19.1)	3 (14.3)	4 (19.1)

Total *N* (%)	21 (100)	21 (100)	19 (90.5)	15 (71.4)	16 (76.2)	6 (28.6)	10 (47.6)
